# Understanding the Mechanism of Atovaquone Drug Resistance in *Plasmodium falciparum* Cytochrome b Mutation Y268S Using Computational Methods

**DOI:** 10.1371/journal.pone.0110041

**Published:** 2014-10-15

**Authors:** Bashir A. Akhoon, Krishna P. Singh, Megha Varshney, Shishir K. Gupta, Yogeshwar Shukla, Shailendra K. Gupta

**Affiliations:** 1 Department of Bioinformatics, Systems Toxicology Group, CSIR-Indian Institute of Toxicology Research, Lucknow, India; 2 Interdisciplinary Biotechnology Unit, Aligarh Muslim University, Aligarh, India; 3 Department of Bioinformatics, Biocenter, Am Hubland, University of Würzburg, Würzburg, Germany; 4 Department of Proteomics, CSIR-Indian Institute of Toxicology Research, Lucknow, India; 5 Academy of Scientific and Innovative Research (AcSIR), New Delhi, India; Institut de Recherche pour le Développement, France

## Abstract

The rapid appearance of resistant malarial parasites after introduction of atovaquone (ATQ) drug has prompted the search for new drugs as even single point mutations in the active site of Cytochrome b protein can rapidly render ATQ ineffective. The presence of Y268 mutations in the Cytochrome b (Cyt b) protein is previously suggested to be responsible for the ATQ resistance in *Plasmodium falciparum* (*P. falciparum*). In this study, we examined the resistance mechanism against ATQ in *P. falciparum* through computational methods. Here, we reported a reliable protein model of Cyt bc1 complex containing Cyt b and the Iron-Sulphur Protein (ISP) of *P. falciparum* using composite modeling method by combining threading, *ab initio* modeling and atomic-level structure refinement approaches. The molecular dynamics simulations suggest that Y268S mutation causes ATQ resistance by reducing hydrophobic interactions between Cyt bc1 protein complex and ATQ. Moreover, the important histidine contact of ATQ with the ISP chain is also lost due to Y268S mutation. We noticed the induced mutation alters the arrangement of active site residues in a fashion that enforces ATQ to find its new stable binding site far away from the wild-type binding pocket. The MM-PBSA calculations also shows that the binding affinity of ATQ with Cyt bc1 complex is enough to hold it at this new site that ultimately leads to the ATQ resistance.

## Introduction

Studies revealed that human malaria is caused by protozoan parasites of the genus *Plasmodium*. The four most common *Plasmodium* species that infect human are *P. vivax*, *P. ovale*, *P. malariae*, and *P. falciparum*. Additionally, a fifth one *P. knowlesi* has also been identified as responsible for infection in human [1] often in many countries of Southeast Asia [2]. According to the latest World malaria report (2012) by World health organization, there were about 219 million cases of malaria in 2010 and an estimated 660 000 deaths. *P. falciparum* predominates in Africa and is the most deadly form leading to death due to malaria. 90% of malaria occurs in Africa and among which 85% deaths happen in children under the age of 5 [3].

*Plasmodium* species can acquire drug resistance through several mechanisms, like change in drug permeability, increased expression of the drug target, or changes in the enzyme target [4]. ATQ drug acts against malarial parasites by inhibiting mitochondrial electron transport [5] and collapsing mitochondrial membrane potential [6]. Based on its structural similarity to ubiquinol, it has been postulated that ATQ binds to parasite Cyt b protein [7]. It is supported by experimental findings that mutations at 268^th^ position in *P. falciparum* Cyt b are unambiguously associated with acquired ATQ resistance [8]. However, the mechanism of ATQ resistance is still not well understood. Thus, there is an urgent need to develop novel disease management strategies against various *Plasmodium sp* induced malaria.

In several studies [9]–[11], researchers have modeled some *P. falciparum* mutations including Y268S using *in silico* methods, however none of them have completely modeled the *P. falciparum* Cyt bc1 complex rather they rely on the ATQ-bound yeast Cyt bc1 complex. Moreover, none of the study has examined the dynamics of the Cyt bc1 ATQ-bound complex. Therefore in the present study, we exploited the *in silico* approaches to identify molecular basis of ATQ drug resistance in the Y286S mutation model of Cyt b protein of *P. falciparum*. To best of our knowledge, this is the first study to report the modeling and molecular dynamics simulation of ATQ-bound *P. falciparum* Cyt bc1 complex in both wild and mutant-type models for nanoseconds time scale.

## Materials and Methods

### Computational model building and quality assessment

The ubiquinol oxidation (Qo) site of the Cytochrome bc1 complex serves as a pocket for ATQ binding [11] and two subunits of the complex (Cyt b and ISP) are involved in ATQ binding [11], [12]. To model the whole complex, amino acid sequences of *P. falciparum* Cyt b (Genbank accession no: NP_059668.1) and ubiquinol-Cyt C reductase ISP subunit (Genbank accession no: XP_001348547.1) were retrieved from the Entrez protein database available at NCBI (http://www.ncbi.nlm.nih.gov). In the process of protein modeling, we observed that no single template was able to satisfy ∼100% query coverage. Hence, the composite modeling which combines various techniques such as threading, *ab initio* modeling and atomic-level structure refinement approaches [13]–[16] implemented in the iterative threading assembly refinement (I-TASSER) server was preferred to build the full-length protein structure of both the protein chains. I-TASSER generates 3D atomic models from multiple threading alignments and iterative structural assembly simulations. The full methodology of the server has been described elsewhere [17]. The template modeling score (TM-score) calculation [18] was used to assess the structural similarity of model and template protein structures [Eq. i].
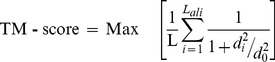
(1)where *L* is the length of the target protein, *L_ali_* is the number of the equivalent residues in two proteins, *di* is the distance of the *i^th^* pair of the equivalent residues between the two structures, which depends on the superposition matrix; the ‘max’ means the procedure to identify the optimal superposition matrix that superposition matrix that maximizes the sum in Eq. i. The scale *d_0_ = ^3^*√*(L – 15) – 1.8d* is defined to normalize the TM-score in a way that the magnitude of the average TM-score for random protein pairs is independent on the size of the proteins.

Confidence score (C-score) was taken into consideration to determine the accuracy of the predicted structure. The score is defined based on the quality of the threading alignments and the convergence of the I-TASSER's structural assembly refinement simulations [Eq. ii].
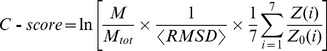
(2)


Where *M* is the number of structure decoys in the cluster and *M_tot_* is the total number of decoys generated during the I-TASSER simulations. <RMSD> is the average RMSD of the decoys to the cluster centroid. *Z(i)* is the Z-score of the best template generated by i^th^ threading in the seven LOMETS programs and *Z_0_(i)* is a program-specified Z-score cutoff for distinguishing between good and bad templates.

The geometry of the theoretical model was improved by side-chain geometry optimization using the ChiRotor algorithm [19]. The modeled structures were further subjected to energy minimization followed by model quality estimation. In order to further design the Cyt b-ISP complex from the individually modeled structures of Cyt b and ISP subunits from *P. falciparum*, we superimposed them to the *S. cerevisiae* bc1 complex already available in Protein Data Bank (pdb entry: 3CX5). From this superimposed structure, we got the coordinates of modelled Cyt b and ISP subunits of *P. falciparum* in the orientation similar to the one found in Cyt bc1 complex of *S. cerevisiae*. Since the yeast bc1 complex also strongly interacts with water molecules in the vicinity of Glu 272 [20], the water molecule was also added to the modeled *P. falciparum* Cyt b-ISP complex before performing the docking experiments.

### Mutation mapping of Cyt b protein at 268^th^ position

All the known point mutations observed at position 268 of *P. falciparum* Cyt b were individually incorporated in the modeled 3D protein structure to scan their impact on ATQ binding. For this, all the mutant models of the Cyt b protein of *P. falciparum* were generated using the mutational modeling protocol of DS3.1. The Build Mutants protocol mutates selected residues to specified types and optimizes the conformation of the mutated residues and their neighbors using MODELER program.

### Retrieval of ATQ structure and modeling of Cyt bc1 complex

PubChem Compound, one of the linked databases within the NCBI's Entrez information retrieval system was accessed for the retrieval of ATQ structure. Both the protein and ligand molecules were prepared before being subjected to docking analysis using Prepare Protein and Prepare Ligand protocols of DS3.1 respectively. Prepare Protein protocol rectify the protein for various problems, such as missing atoms in incomplete residues; missing loop regions; alternate conformations (disorder); nonstandard atom names; incorrect protonation state of titratable residues etc. The generation of 3D conformation of ATQ was attained by the Prepare Ligands tool.

Molecular docking experiments of the ATQ into the Qo site of both mutated and non-mutated variants of the Cyt b protein was performed by CDOCKER, a molecular dynamics (MD) simulated-annealing-based algorithm [22]. The ATQ was assumed to bind in the same binding pocket to that of ligand stigmatellin, a known inhibitor of Qo site of the Cyt bc1 complex. Water molecules were removed, except HOH7187 which has been reported to play important role in the observed hydrogen bonding network [9]. General-purpose all-atom force field (CHARMm) with a wide coverage for proteins, nucleic acids and general organic molecules was included in the random structure generation. 10 orientations were generated for the ATQ, improved by performing simulated annealing method and finally refined by applying low, but most accurate full potential as a refined pose minimization method.

### Molecular dynamics simulations

Molecular dynamics simulations were performed with Gromacs ver. 4.5.3. The ligand topology and parameterization was attained with SwissParam (http://www.swissparam.ch/), an automatic tool that generates topology and parameters based on the Merck molecular force field. The wild and mutant-type Cytbc1 complexes (Cyt b-ISP/ATQ) were subjected to molecular dynamics simulation with explicit TIP3P water solvation model in the Isothermal–isobaric (NPT) ensemble using the AMBER99SB force field. Each system was minimized with the steepest descent method to relax unfavorable contacts between molecules and equilibrated for 150 ps before production runs to achieve stability during production dynamics. Simulations were performed at a constant temperature of 310 K and pressure of 1 atm, using Particle Mesh Ewald method [23] for long-range electrostatic [24] and van der Waals (vdW) [25], [26] interactions with a cut-off of 1.4 nm while constraints were applied on all bonds using the LINCS [27] algorithm. All systems were simulated in the NPT (fixed number of atoms N, pressure P, and temperature T) ensemble using the v-rescale coupling algorithm [28] and the Parrinello–Rahman coupling algorithm [29] for 90 ns and time step of 2 fs without any position restraints.

### MM/PBSA calculations

The MM/PBSA approach [30] was applied to perform the binding free energy calculations. The binding free energy of a protein to a ligand (ΔG_bind_) is defined from the complex, protein and ligand free energies (G_complex_, G_protein_ and G_ligand_, respectively) as [Eq. iii]
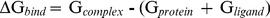
(3)


Each free energy term is obtained from a MD-derived ensemble of structures as the sum of six terms as mentioned in [Eq. iv].
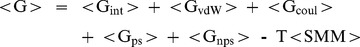
(4)


Where G_int_, G_vdW_ and G_coul_ indicate the internal (including bond, angle, and torsional angle energies), van der Waals and coulombic energy terms, respectively, collectively defined “gas phase terms”. <G_ps_> and <G_nps_> are the polar and nonpolar solvation energy terms, respectively. <SMM> is the entropic term. Angle brackets denote the average along the structures.

The single trajectory method (STM) has been used for both the wild-type and the mutant systems. The STM requires the trajectory of the complex to be run only. The structures of the free forms of the protein and ligand species were obtained by stripping the partner molecule from the structure of the complex. Thus, zeroing out the <G_int_> term in the STM analysis.

For the MM/PBSA calculations, the GMXAPBS tool was used [31]. In particular: (1) the van der Waals term was calculated with Gromacs, (2) the coulombic term was calculated using the APBS accessory program coulomb, (3) the polar solvation term was calculated via APBS [32], using the non-linearized Poisson Boltzmann equation. Internal and external dielectric constants were set to 1 and 80, respectively; temperature was set to 310 K; the salt concentration was defined as 0.15 M; grid spacing was set to an upper limit of 0.5 Å, (4) The nonpolar solvation term was considered proportional to the solvent accessible surface area (SASA) as shown in [Eq. v]
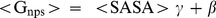
(5)where γ = 0.0227 kJ mol^−1^ Å^−2^ and β = 0 kJ mol^−1^ [33]. The dielectric boundary was defined using a probe of radius 1.4 Å.

The equilibrium phase (70–90 ns) of the two molecular dynamics simulations (i.e., 151 equally time-distant frames for each system) was considered for MM/PBSA calculations. The standard errors (SE) were calculated as [Eq. vi]
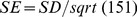
(6)where SD is the standard deviation.

### Principal component analysis

The Principal Component Analysis (PCA) was used to characterize and compare the overall motions of the two complexes. We calculated the principal components on the converged simulation and focused on the movement of the 731 Cα atoms of the protein that resulted in 2193 dimensional displacement vectors. The 2193×2193 covariance matrix was then diagonalized to obtain its eigenvalues and eigenvectors. The PCA method decomposes the overall protein motion into a set of modes (eigenvectors) that are ordered from largest to smallest contributions to the protein fluctuations. The contribution of atom j to the i^th^ mode's fluctuation was obtained using the following equation [Eq. vii]:
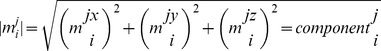
(7)


The 
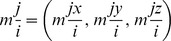
 term represents the component vectors of the j^th^ atom for the i^th^ mode.

Each of the eigenvectors depicts a collective motion of particles and their respective amount of participation is represented by eigenvalues. Usually, the first ten eigenvectors are sufficient to describe almost all of the conformational subspace accessible to the protein.

## Results and Discussion

The Y268 residue of Cyt b in *Plasmodium* is known to play a key role in ATQ drug resistance [8] and thus, can be used as a potential resistance marker [34]. Studies of *P. falciparum* resistance to ATQ revealed 3 point mutations at 268^th^ position (Y268N, Y268S, Y268C) [8], [21], [35]. The substitution of one or several amino acid residues in a protein often lead to substantial changes in properties such as thermodynamic stability, catalytic activity, or binding affinity [36]–[38]. As point mutations at Y268 have already been identified for ATQ resistance, it is obvious that this substitution should affect the fitting and binding of the drug. ATQ drug, an analogue of coenzyme Q (ubiquinone), interrupts electron transport and leads to loss of the mitochondrial membrane potential [6].

### Molecular modeling of Cyt b-ISP complex of *P. falciparum*

The 3D structure details of proteins are of major importance in providing insights into their molecular functions. Since computational methods not only help in directing the selection of key experiments, but also in the formulation of new testable hypotheses [39]. Therefore, in the absence of X-ray structures, the 3D theoretical models were built using the I-TASSER. I-TASSER [12], [13], a hierarchical protein structure modeling approach based on 2 protein structure prediction methods i.e., threading and *ab initio* prediction, was used to build 3D models of the Cyt b and ISP sequences from *P. falciparum*. I-TASSER uses restraints from templates identified by multiple threading programs to build full length model using replica-exchange Monte-carlo simulations. Cyt b chain was modeled by I-TASSER using restraints from PDB templates 2IBZ, 3CX5, 1EZV, 1BCC and 3H1J while the ISP subunit used restrains from PDBs 1KB9, 3CX5, 1EZV, 1BCC, 3CWB, 1BGY and 3L72. No significant similarity was observed with any of the PDB templates against N-terminal sequence of ISP, therefore, this part was modeled by I-TASSER using ab-initio approach. Although, including the long N-terminal sequence, modeled by ab-initio method can compromise the overall model accuracy, in the present study, we used full length model because of multiple reasons, (i) N-terminal 158 residues (modeled) of ISP subunit may also contribute in the reliable folding of tertiary structure as we observed several secondary structure elements in that region (Figure S1). Several studies have shown that the removal of N-terminal building blocks from the structure may contribute in error during protein folding [40]–[42] as the protein may acquire a non-native stable conformation due to mis-association of the adjunct building blocks. (ii) After modeling of the Cyt b-ISP complex, we observed the N-terminal sequence of chain moves back and forth over the active-site cleft (Figure S2) therefore this part was also taken into consideration. (iii) Since, even a single amino acid residue may significantly affect the conformation of binding site if present around 4.5 Å radius of the ligand, and may also alter the binding efficacy [43], thus we preferred to include this region in our model, so that the possible impact of N-terminal sequence in ATQ binding may be evaluated during simulations.

The computation of a structural alignment of 2 protein structures is critical in modeling, as in contrast to sequence alignment methods, structure alignment methods aim directly on optimizing the structural similarity of the input proteins [44]. The implemented TM-score in I-TASSER is a sensitive scale to the global topology for measuring the structural similarity between 2 proteins. Statistically, a TM-score <0.17 means a randomly selected protein pair with the gapless alignment taken from PDB. The better TM-score of our Cyt b model (0.99±0.04) and ISP subunit model (0.35±0.12) indicates much better structural match of the target sequence with the templates. I-TASSER provides C-score to estimate the quality of the predicted models, and is calculated based on the significance (i.e. Z-score) of the threading alignments in LOMETS and the convergence parameters (i.e. cluster density) of the I-TASSER structure assembly simulations. The C-score scheme has been extensively tested in large-scale benchmarking tests [13], [18] and is typically in the range (−5, 2), where a higher score reflects a model of better quality. The C-score of the best predicted model of Cyt b and ISP subunit model was +2.00 and −3.25.

The Side-Chain Refinement protocol of DS was used to optimize the protein side-chain conformations. This protocol uses ChiRotor algorithm and CHARMm force field to systematically search for optimal side-chain conformation of all residues and generates a model structure with the best side-chain conformation. Model quality estimation is critically important in computational protein modeling, since the accuracy of a model determines its suitability for specific biological and biochemical experimental design [45]. The fitness of a protein sequence in its current 3D environment before and after side chain refinement was evaluated by Verify Protein (Profiles-3D). The Verify score of the protein is the sum of the scores of all residues in the protein and has been used by several researchers for structural assessment of theoretical models [46]–[48]. If the overall quality is lower than the expected low score, the structure is certainly misfolded. The verify score of the Cyt b model before and after energy minimization was 89.2 and 93.5, with the expected low score of 77.1, showing that the structure after side chain refinement was much better than the non-refined one. Similarly, we observed the verify score of the ISP model as 89.4, with the expected low score of 72.7906. The exact percentage of amino acids located in the core region was calculated by Procheck program. Generally, the atomic resolution structures have over 90% of their residues in the most favorable regions and for lower resolution structures resolved at 3.0–4.0 Å, the core percentage is around 70% [49]. The Ramachandran plot showed that 90.3% residues were located in the core region of Cyt b chain and 83.33% residues were in the core region of ISP chain, indicating the reliability of models for further studies (Figure S3).

Moreover, we also looked into the RMSD of the modelled Qo site and the templates chosen for modeling of wild-type protein of *P. falciparum*. The calculated residuals for 1EZV, 2IBZ, 3CX5, 3H1J and 1BCC templates from our modeled protein were 0.48 Å, 0.44 Å, 0.46 Å, 0.56 Å and 1.02 Å respectively. The insignificant RMSD of Qo site from the respective templates further supports the model accuracy.

### Mutagenesis of Cyt b protein

Most of the reported mutations in Cyt b either destabilize the important hydrophobic interactions between ATQ and the amino acid residues in the binding site of the protein, or are responsible for the change of pocket volume [9], [50]. The conserved bulky Tyrosine (T) residue at 268^th^ position forms hydrophobic contact with the ATQ drug in the Qo region of the ubiquinol oxidation site. Substitution of the hydrophilic and less bulky asparagine (N) at position 268 not only reduces the volume of the binding pocket but it also decreases the affinity and binding of ATQ [51]. Besides, substitution of serine (S), a hydrophilic amino acid, limits hydrophobic contact with ATQ resulting in marked decrement of ATQ susceptibility in mutated malaria parasites [21], [35]. Moreover, a role for cysteine (C) in impairment of ATQ binding has also been observed [8]. Hence, *P. falciparum* Cyt b protein mutations (Y268S, Y268N and Y268C) were implemented in the 3D structure of the parent model using mutational modeling protocol of DS.

### Active site selection and docking calculations of ATQ

**Figure 1:**
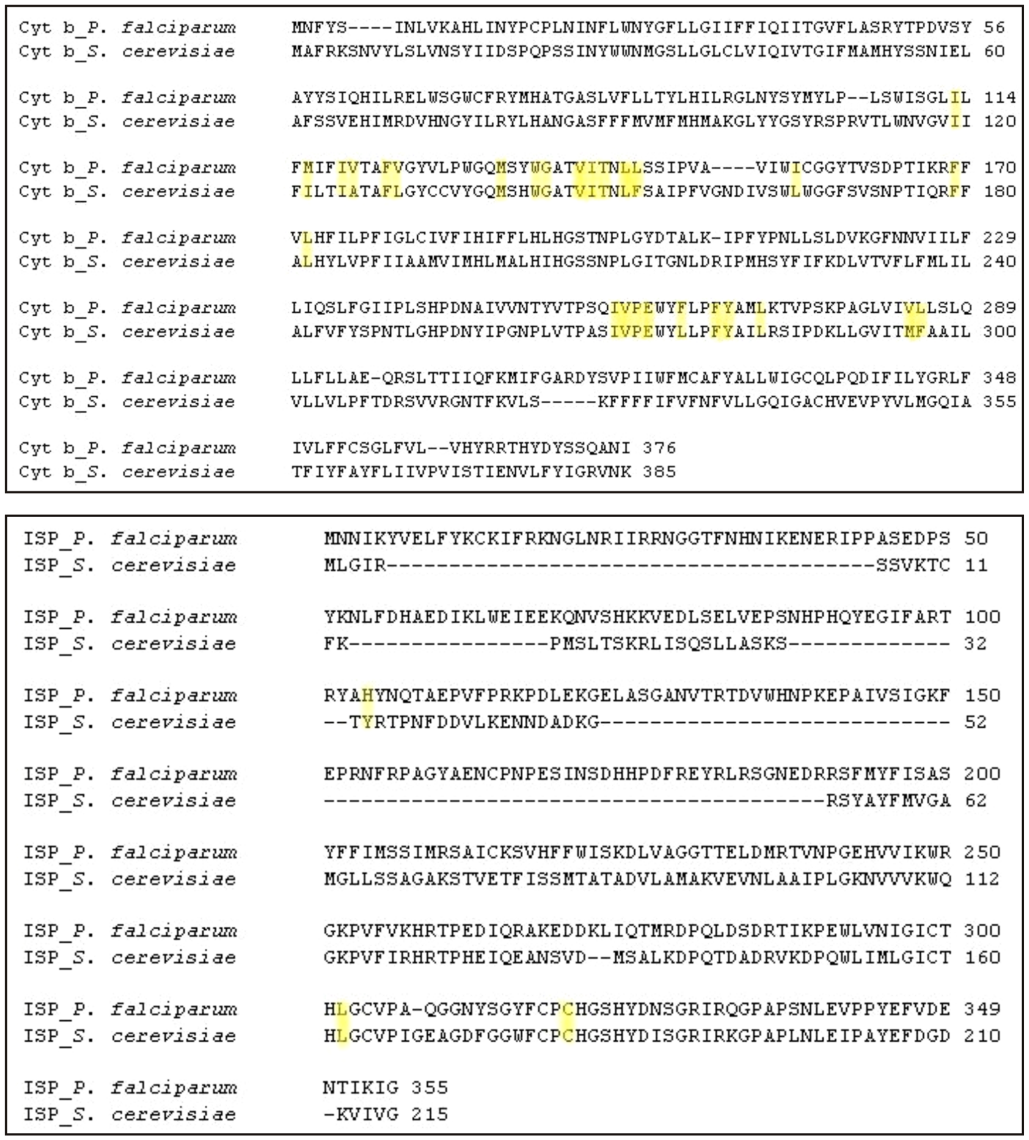
Sequence alignment of Cyt b protein and ISP chain of *P. falciparum* and *S. cerevisiae*. Amino acid residues involved in the formation of Qo site are highlighted in yellow tinted color.

**Figure 2:**
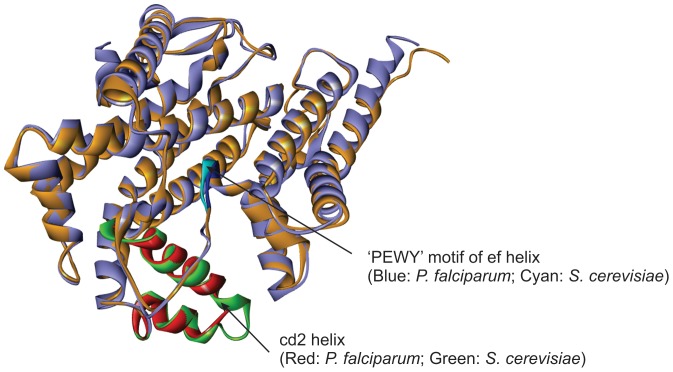
Structural overlay of the homology model of Cyt b protein of *P. falciparum* (blue) with the Cyt b unit of *S. cerevisiae* (golden) (PDB ID: 3CX5). A total of 4 amino acid residues deletion in cd2 helix (red) of *P. falciparum* resulted in structural displacement when compared with the same domain of *S. cerevisiae* (green). Also the structural changes in ‘PEWY’ motif of ef helix are shown.

ATQ is very likely to bind in a manner similar to stigmatellin, a known inhibitor of Qo site of the Cyt bc1 complex [10] and hence the potential binding site for stigmatellin as proposed by Solmaz and Hunte (PDB acquisition code 3CX5) [20] was chosen as the biologically favorable site for ATQ docking. After checking the conservancy of the active site residues of Cyt bc1 complex of *P. falciparum* (CYTB: MET116, ILE119, VAL120, PHE123, VAL124, MET133, TRP136, GLY137, VAL140, ILE141, THR142, LEU144, LEU145, ILE155, PHE169, LEU172, ILE258, VAL259, PRO260, GLU261, PHE264, PHE267, TYR268, LEU271, VAL284, LEU285; ISP: HIS104, LEU302, CYS319) with the Cyt b protein of *S. cerevisiae*, we observed that with the exception of 8 amino acid residues, all other residues were conserved between these 2 species (Figure 1). Moreover, we observed the non-identical residues of *P. falciparum* were also showing strongly similar properties (scoring>0.5 in the Gonnet PAM 250 matrix) with the amino acid residues present in *S. cerevisiae*. *Plasmodium* Cyt b is unusual in the sense that cd2 helix (a critical structural component of the catalytic Qo site) contains a 4-residue deletion that is not found in non-Apicomplexan sequences. It would seem very likely that this would alter the fold of the Qo site when compared to the Cyt b structural data available in the PDB. Therefore, we aligned the 3D structures of Cyt b from *P. falciparum* and *S. cerevisiae*. The structural overlay of the homology model of the *P. falciparum* Cyt b with the yeast Cyt b has been presented for comparison in Figure 2. Our model suggests that the 4 residue deletion in the cd2 helix results in a 0.83 Å displacement of this structural element compared with the yeast Cyt b (Figure 2). Likewise catalytically essential ‘PEWY’ motif of the ef helix was observed to be displaced by 0.35 Å from the yeast enzyme. To perform docking analysis, the ATQ structure was modeled properly using the Prepare/Filter Ligand tool of DS. Docking is a potentially powerful and inexpensive method for the discovery of binary interactions. The Qo site residues were chosen to define the binding site in our modeled Cyt bc1 complex of *P. falciparum* based on known Qo site inhibitor interactions for *S. cerevisiae* available in protein data bank (pdb id: 3CX5, 2IBZ). A total of 10 random ligand conformations were generated from the ATQ structure through high temperature molecular dynamics, followed by random rotations. These conformations were refined by grid-based (GRID 1) simulated annealing and a final full force field minimization method. We observed that ATQ was showing less binding affinity towards all the mutant variants when compared to the wild-type.

**Figure 3:**
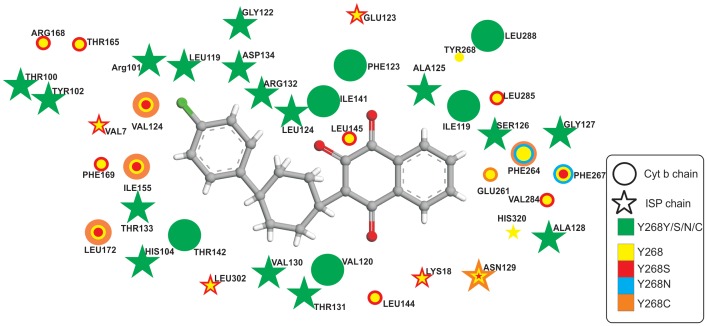
Two dimensional contact plots of amino acid residues from the wild and all screened mutant models of Cyt bc1 complex from P. falciparum in the vicinity of 5 Å radius around ATQ. It may be noted that in the mutant models the position 268 shifted away from the 5 Å radius of ATQ Binding site. Whereas green color indicates that the particular amino acid residue is present in the wild as well as in all mutant models in the observed area; yellow, red, blue, cyan color shows amino acid residues present only in wild type, Y268S, Y268N and Y268C mutant models respectively.

While examining all the contact amino acid residues within 5 Å of the ATQ, as shown in the Figure 3, we observed the presence of Y268 within the ATQ binding site in the wild-type protein. Surprisingly when Y268 was mutated to any of the 3 possible amino acid mutations, i.e.Y268N, Y268S, Y268C, this position shifted far away from ATQ binding site. We feel the shift of amino acid residue at 268^th^ position after point mutation might be the main reason of ATQ resistance in the mutant models. In order to understand the mechanistic insight of ATQ resistance in the mutant models, we further performed detailed molecular dynamics simulation studies by considering only the most prevalent mutant variant (Y268S) identified in various experimental settings [9].

### Dynamic insights into the Cyt bc1 modeled complexes

**Figure 4:**
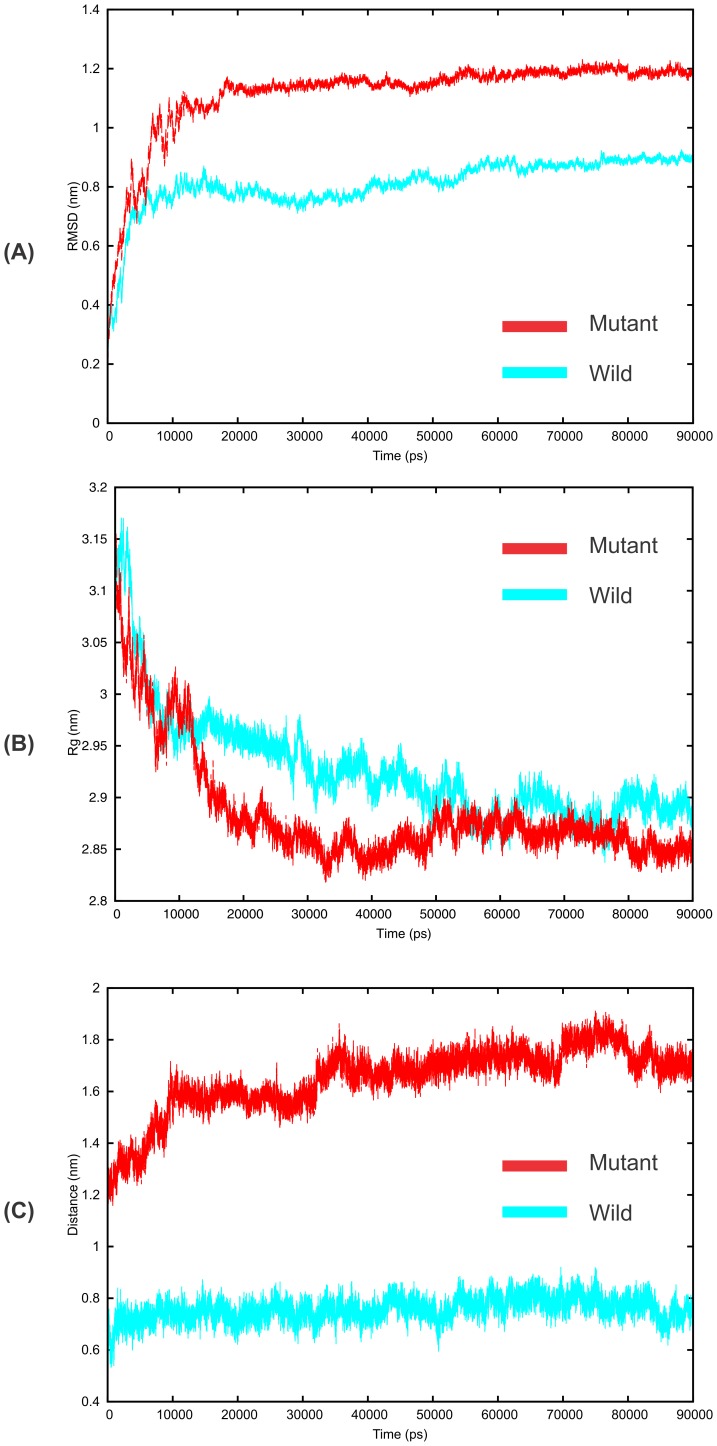
Molecular dynamics simulation graph of wild (Y268) and mutant (Y268S) Cyt bc1 complexes of *P. falciparum* in solution. (A) Root mean square deviation (RMSD) of backbone atoms with respect to their initial complexes over a period of 90 ns simulation time. (B) Radius of gyration (Rg) graph and (C) Distance of ATQ from the Qo site over the whole simulation in wild as well as mutant type.

Several simulation studies have already shown nice correlation between computational and experimental measurements of macromolecular dynamics [52]–[55]. As molecular dynamics based techniques can provide more precise protein–ligand models in the state close to natural conditions therefore to get detailed insights into the molecular basis of ATQ resistance in malaria, we individually simulated ATQ-bound wild-type and the most prevalent mutant variant (Y268S) of *P. falciparum* Cyt b protein [9] till we attained the convergence of MD simulation around 70–90 ns of production run. To explore the dynamic stability of the systems, root mean square deviation (RMSD) of the Cyt b-ISP backbone atoms (both wild-type and mutant complexes) was computed with reference to their respective initial structures as a function of simulation run time. MD simulation shows that the wild-type complex undergoes less structural changes when compared with the mutant complex over a period of 90 ns simulation time (Figure 4A, in Cyan) and RMSD of both the complexes remain almost stagnant after ∼65 ns time period. The plotted graph in Figure 4A (red) shows that although the RMSD of mutant complex was initially in agreement with the wild-type complex but the complex underwent significant deviation after 7ns and reaches at ∼1.1 nm RMSD (consistent throughout the whole dynamics run), showing the comparatively unstable behavior of the mutant complex. We further extended our study to analyze the compactness (radius of gyration) of both the wild-type and mutant complexes. Though we did not find much significant difference in Rg values, however we noticed that at the equilibrium state mutant structure was more compact than the wild-type (Figure 4B). We were also interested to check the ATQ distance from the Qo site during the entire dynamics run. After analyzing the results, we noticed that ATQ retained its position throughout the whole dynamics run in the wild-type however it showed significant fluctuations during the entire 90ns simulation in mutant case (Figure 4C). Even as clear from the Figure 4C, the distance between the Qo site and ATQ was increasing with respect to time. We may attribute this behavior to the change in binding pocket configuration and also because of some steric clashes of ATQ near the binding site.

**Figure 5:**
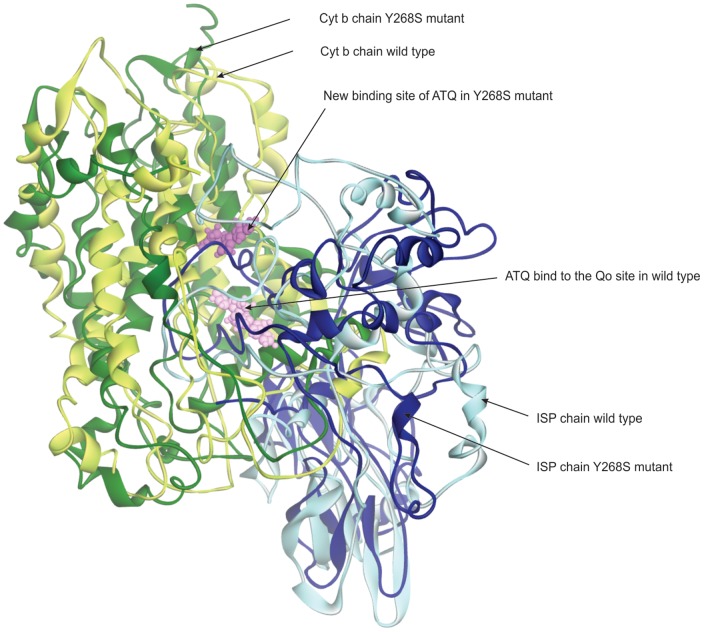
ATQ binding site in the wild and mutant (Y268S) Cyt bc1 complex of *P. falciparum*. The figure indicates that ATQ binds to a new site in the mutant model which is around 12 Å distant from the Qo site. The structure was captured from the average structure of the Cyt bc1 complex of *P. falciparum* over 70–90 ns (converged part of the trajectory).

In order to understand how the resistant mutations affect the interaction of ATQ in mutant protein, we calculated root mean square inner product (RMSIP) from both the complexes to ascertain the convergence of conformational sampling instep-wise manner till we find the convergence. In this process, we increased the production run from initial 20 to 90 ns in the block of 10 ns. In general, RMSIP values between 0.5–0.7 represent adequate convergence [56] and here in our case, we observed an acceptable convergence measure of 0.55 from 70–90 ns trajectory. Therefore, this part of simulation was used for the computation of average structure of the complex. To remove the crudeness of the average structure, the structure was subjected to 1000 steps of energy minimization using the Smart Minimizer (SM) available in DS. SM begins with the Steepest Descent method, followed by the Conjugate Gradient method for faster convergence towards a local minimum. We observed the ATQ interactions were predominantly hydrophobic, although certain hydrophilic interactions exist temporarily during the MD simulation. Histidine residue 181 in the ISP (Yeast) is reported to form a strong hydrogen-bond with certain classes of Qo-bound inhibitors such as stigmatellin ('b-distal' inhibitors). We noticed that in *P. falciparum*, HIS104 of ISP chain forms such stable interaction with the ATQ. However, we did not find the stability of the ATQ hydrogen bond that was supposed to be formed via a water molecule with Glu (Glu-272 in yeast) of Cyt b. In mutant case, both these interactions were altogether absent. In the mutant model irrespective of ATQ binding at Qo site, it was found to get stabilized at a new site (site II) which is around 12 Å apart from the Qo site. The two different binding modes of ATQ in wild and mutant-type are shown in Figure 5. Moreover, we also noticed that the induced mutation makes the active site to undergo significant conformational changes that reduce both the active site volume (7174.6 Å^3^) and its surface area (4783.4 Å^2^) than the wild-type (11388 Å^3^; 7269.3 Å^2^). The shrinkage in both volume and surface area (also reflected by Rg values, Figure 4C) might be also one of the probable reasons for different binding site selectivity of ATQ in mutant-type. Overall, all the above consequences might be the causing agents for ATQ to have different selectivity in the mutant protein than the wild-type.

### MD-based binding free energy calculations of ATQ-bound Cyt bc1 complex

To acquire an estimate of the binding free energies in the two systems (i.e., Y268 and Y268S) and to inspect the differences in terms of polar and non-polar interactions, we performed binding free energy calculations on the converged MD trajectories (70–90 ns) using the MM/PBSA approach. For this purpose, we took advantage of the GMXAPBS tool [30]. Here we would like to mention that in this investigation we were interested in highlighting the differences of binding free energies of similar complexes. As already reported for several other investigations [30], [57], [58], we will thus ignore the entropic term and focus only on the binding enthalpy, defined as the sum of 4 terms, namely coulombic, van der Waals, polar solvation and non-polar solvation. The results of our calculations are shown in Table 1.

**Table 1:** MM/PBSA binding free energies (kJ/mol) of wild-type and mutant Cyt bc1/ATQ complexes.

Binding free energies (kJ/mol)	Y268S	Y268
**ΔG_coul_** 1	−71.438±1.993	−49.334±1.527
**ΔG_vdW_** 2	−188.381±1.118	−209.546±1.023
**ΔG_ps_** 3	190.650±2.357	246.728±2.430
**ΔG_nps_** 4	−17.924±0.070	−19.962±0.037
**ΔG_polar_** 5	119.212	197.394
**ΔG_nonpolar_** 6	−206.305	−229.508
**ΔG_bind_** 7	−87.094±2.178	−32.113±2.653

1 : coulombic term;

2 : van der Waals term;

3 : polar solvation term;

4 : nonpolar solvation term;

5 : polar term (sum of coulombic and polar solvation terms);

6 : nonpolar term (sum of van der Waals and nonpolar solvation terms);

7 : computational binding free energy.

Both systems present a similar pattern: the van der Waals, coulombic and non-polar solvation terms are negative, meaning that they favor the formation of the complex. On the contrary, the polar solvation term is positive, which indicates that it antagonizes the binding process. This observation might be due to the cost of desolvating the polar moieties in the protein residues and in the ligand. It is noteworthy that in both cases the polar contribution, defined as the sum of the coulombic and the polar solvation terms, is positive. Overall, the MM/PBSA analysis reveals that the complex stabilization is promoted by the non-polar contribution only. Such a behavior has been described in the past both for protein-ligand [59] and for protein-protein [31] interactions. The Y268S mutation significantly affects all terms.

Our study demonstrates that the binding of ATQ with the site II (newly identified binding site) of *P. falciparum* Cyt bc1 complex was energetically favorable compare to the site I (Qo site). We assume the sufficient binding energy of ATQ at site II might be preventing ATQ to bind its native site (Qo) thereby resulting in the loss of the anti-malarial efficiency of ATQ in the Y268S mutant-type.

### Essential dynamics analyses of the Cyt bc1 complexes

**Figure 6:**
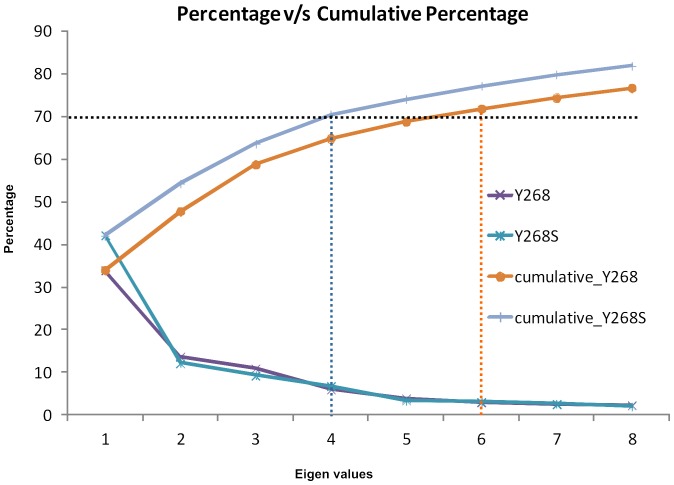
Proportion of variance and cumulative proportion of total variance of first ten eigenvalues of the wild (Y268) and mutant variant (Y268S) of *P. falciparum* proteins.

To support the MD results, we performed essential dynamics study of both the simulated complexes and our results shows that the cumulative variance captured by the first few eigenvectors or principal components of the wild-type complex was comparatively lower than the mutant-type (Figure 6).

The analysis indicates that the Y268S/ATQ complex pertain more motions than the wild-type complex. This is in consistent with the earlier dynamic results where we observed some fluctuations and far away displacement of ATQ from the Qo site in the Y268S/ATQ complex.

## Conclusion

The emerging acquired drug resistance because of mutations has presented a challenge to follow the traditional drug discovery pipelines. Even a single point mutation has potential to produce the drug resistance. In this study, we evaluated the drug-resistance mechanism of ATQ in the mutated (Y268S) Cyt b protein of *P. falciparum* using the potential of *in silico* methods. We observed that the interaction between ATQ and Cyt b is mainly stabilized by the hydrophobic contacts and after mutation of Y268S ATQ contacts with the Qo site are greatly reduced. Such findings have also been reported by other authors [9]. We presume that this reduction in Qo contacts and also change in the volume and surface area of the binding pocket (similar observations have been made by Kessl et al. 2005 [9] in I269M mutation of ATQ-bound yeast bc1 complex) enforce ATQ to find its desirable contacts at distant location from its wild active site. Moreover, the MM-PBSA calculations firmly proved the tighter binding of ATQ with the mutant-type at additional binding site (first time observation), present at ∼12 Å faraway from the active site, thereby raising no choice for ATQ to bind its native site. This might be the probable reason for ATQ anti-malarial efficacy loss in Y268S mutants. We hope the structural details presented in this study would aid the experimental plan to design new suitable selective ligands that could have correct size to fit properly in the active site even after mutation. Such ligands might be able to resist the mutation effect and can be used as future effective drugs against malaria.

## Supporting Information

Figure S1 ISP subunit of Cyt bc1 complex of *P. falciparum* with predicted secondary structure elements. It is important to note that the initial 158 N-terminal residues, which were not present in the *S. cerevisiae* Cyt bc1 complex subunit in PDB file 3CX5, are also involved in critical secondary structure confirmations. This is the reason why we consider full length ISP subunit in our analysis.
(TIF)

Figure S2 The Qo site (ATQ binding site) of Cyt bc1 complex of *P. falciparum* is shown. The N-terminal residues of ISP chain are involved in the formation of active site (Qo) cleft (red color and shown as surface model). Cyt b subunit is shown in green color and ISP subunit as blue.
(TIF)

Figure S3 The Ramachandran plots of modeled Cyt b protein and ISP subunit of *P. falciparum* in Cyt bc1 complex are shown. The plots indicate the quality of the modeled structure was satisfactory.
(TIF)
