# Supplementary figures and images for: Understanding the Mechanism of Atovaquone Drug Resistance in Plasmodium falciparum Cytochrome b Mutation Y268S Using Computational Methods
Source: PLoS One. 2014 Oct 15;9(10):e110041. doi: 10.1371/journal.pone.0110041 (PMC4198183; doi:10.1371/journal.pone.0110041)

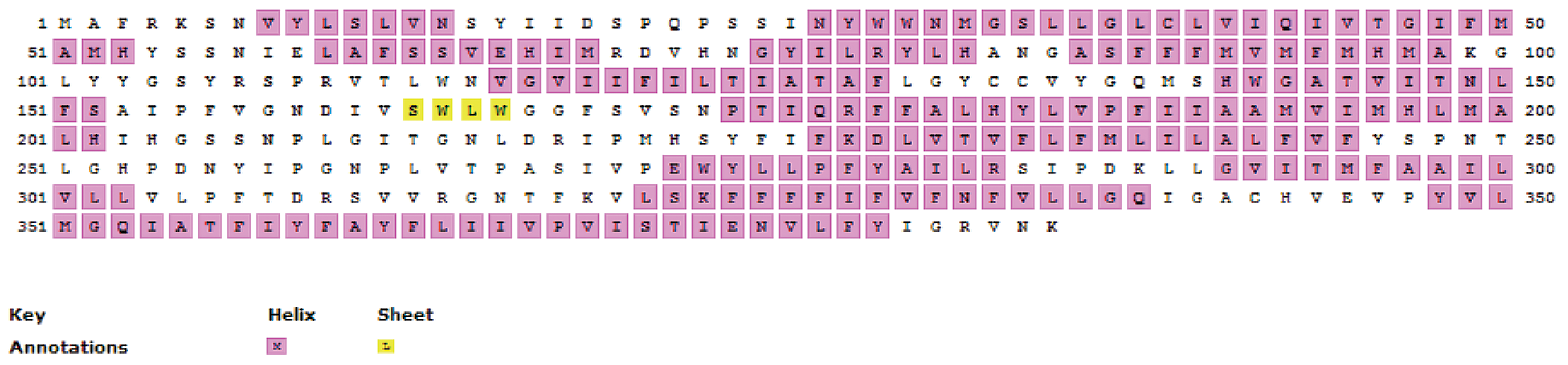

Supplement: Figure S1 — ISP subunit of Cyt bc1 complex of P. falciparum with predicted secondary structure elements. It is important to note that the initial 158 N-terminal residues, which were not present in the S. cerevisiae Cyt bc1 complex subunit in PDB file 3CX5, are also involved in critical secondary structure confirmations. This is the reason why we consider full length ISP subunit in our analysis. (TIF) [file pone.0110041.s001.tif]

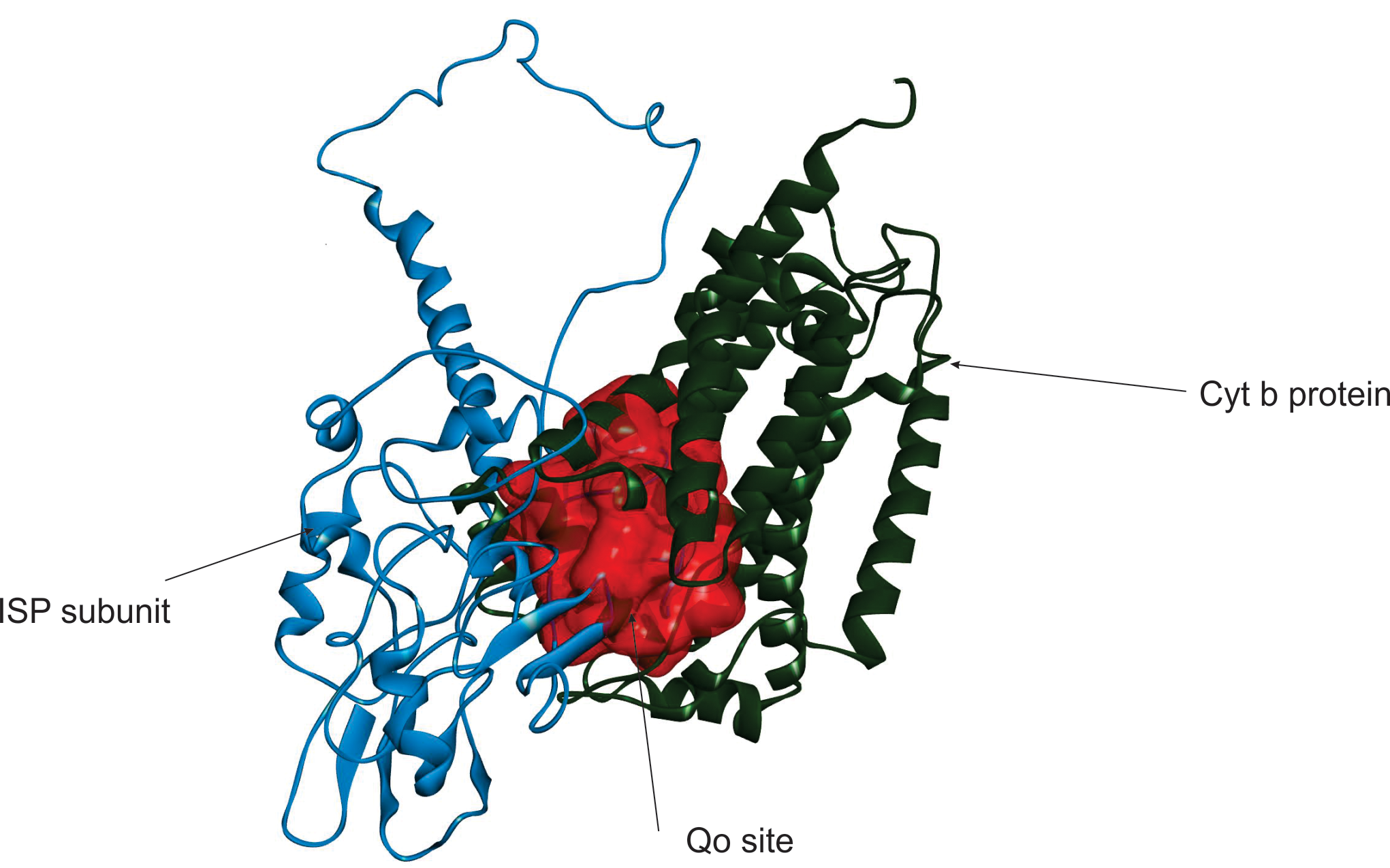

Supplement: Figure S2 — The Qo site (ATQ binding site) of Cyt bc1 complex of P. falciparum is shown. The N-terminal residues of ISP chain are involved in the formation of active site (Qo) cleft (red color and shown as surface model). Cyt b subunit is shown in green color and ISP subunit as blue. (TIF) [file pone.0110041.s002.tif]

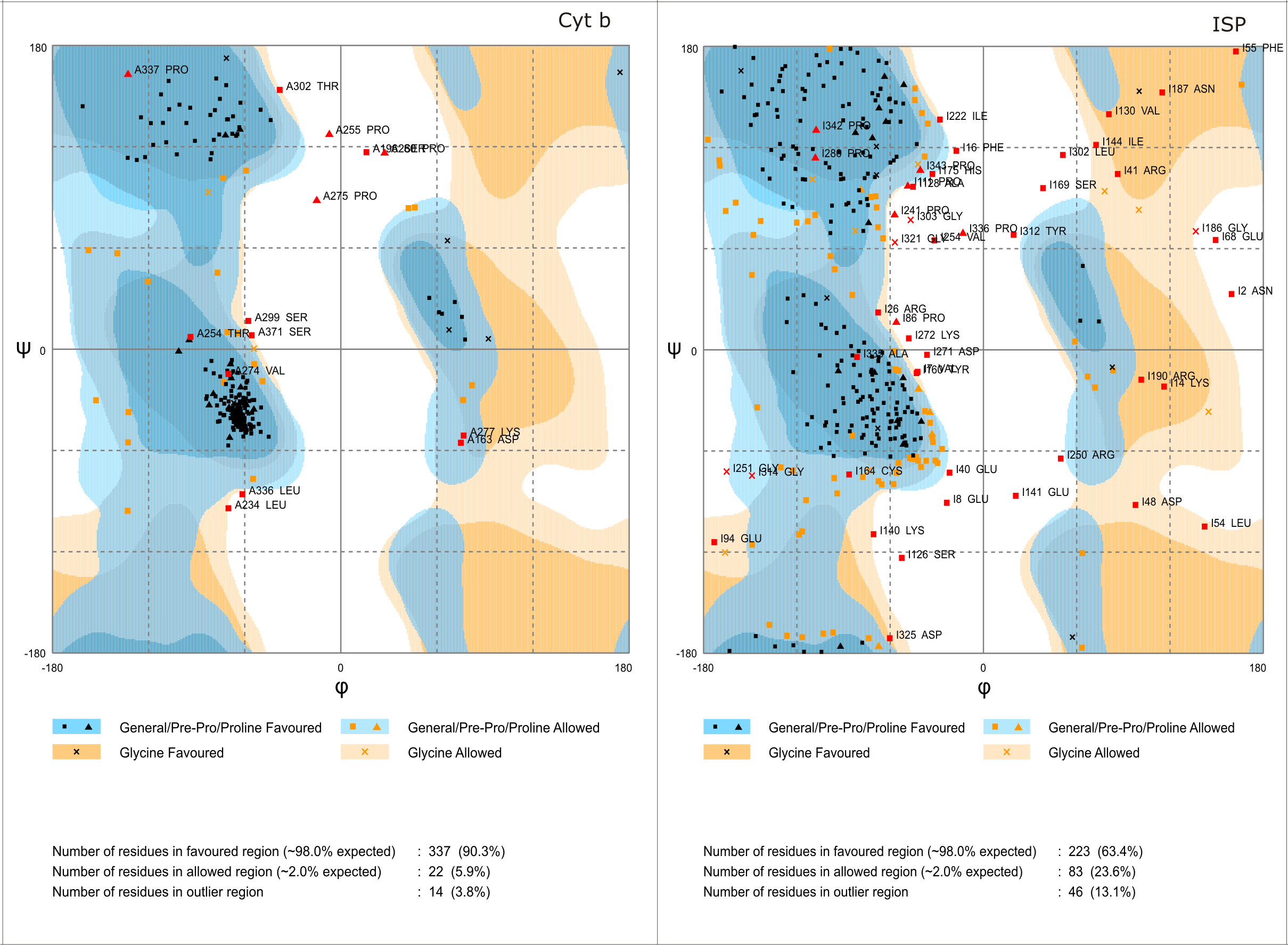

Supplement: Figure S3 — The Ramachandran plots of modeled Cyt b protein and ISP subunit of P. falciparum in Cyt bc1 complex are shown. The plots indicate the quality of the modeled structure was satisfactory. (TIF) [file pone.0110041.s003.tif]
